# Synthetic and Natural Biomaterials in Veterinary Medicine and Ophthalmology: A Review of Clinical Cases and Experimental Studies

**DOI:** 10.3390/vetsci11080368

**Published:** 2024-08-12

**Authors:** Fabio Leonardi, Barbara Simonazzi, Filippo Maria Martini, Pasquale D’Angelo, Ruben Foresti, Maddalena Botti

**Affiliations:** 1 Department of Veterinary Science, University of Parma, 43126 Parma, Italy; fabio.leonardi@unipr.it (F.L.); filippomaria.martini@unipr.it (F.M.M.); maddalena.botti@unipr.it (M.B.); 2 CNR-IMEM, Italian National Research Council, Institute of Materials for Electronics and Magnetism, 43126 Parma, Italy; pasquale.dangelo@imem.cnr.it (P.D.); ruben.foresti@unipr.it (R.F.); 3 Department of Medicine and Surgery, University of Parma, 43123 Parma, Italy; 4 CERT, Center of Excellence for Toxicological Research, 43123 Parma, Italy

**Keywords:** biomaterials, soft material, hard material, veterinary medicine, ophthalmology, systematic review, personalized medicine

## Abstract

**Simple Summary:**

Three-dimensional printing technology is a method of creating a three-dimensional object layer by layer using a computer-generated design. This method has enabled the production of custom models of organs or organ parts, leading to the emergence of “personalized medicine”. The materials used in 3D printing include plastic, metal, and polymers. This review discusses the current state and future prospects of six biomaterials used in veterinary medicine and ophthalmology. Polycaprolactone is suitable for replacing hard tissue defects and is well tolerated in the eye, making it useful for ocular drug delivery devices. Pluronic is used for bone tissue engineering applications and could also be employed for drug delivery in ophthalmology. Silk is used for composite osteogenic scaffolds and vascular grafts, and it may be tested for creating protective lenses for the eye. Collagen is used to produce bioengineered corneas to improve the treatment of corneal ulcers. Alginate is used in cardiac and orthopedic procedures and is also employed in various ocular delivery systems for corneal repair. Hyaluronic acid is commonly used as a lubricant and can serve as a regenerative scaffold during the corneal healing process.

**Abstract:**

In recent years, there has been a growing interest in 3D printing technology within the field of bioengineering. This technology offers the ability to create devices with intricate macro- and micro-geometries, as well as specific models. It has particularly gained attention for its potential in personalized medicine, allowing for the production of organ or tissue models tailored to individual patient needs. Further, 3D printing has opened up possibilities to manufacture structures that can substitute, complement, or enhance damaged or dysfunctional organic parts. To apply 3D printing in the medical field, researchers have studied various materials known as biomaterials, each with distinct chemical and physical characteristics. These materials fall into two main categories: hard and soft materials. Each biomaterial needs to possess specific characteristics that are compatible with biological systems, ensuring long-term stability and biocompatibility. In this paper, we aim to review some of the materials used in the biomedical field, with a particular focus on those utilized in veterinary medicine and ophthalmology. We will discuss the significant findings from recent scientific research, focusing on the biocompatibility, structure, applicability, and in vitro and in vivo biological characteristics of two hard and four soft materials. Additionally, we will present the current state and prospects of veterinary ophthalmology.

## 1. Introduction

Three-dimensional (3D) printing technology can be used to produce biological tissues and organs through a process called bioprinting, which involves printing biochemical material and living cells to create three-dimensional biological structures. This technology is the result of interdisciplinary studies in the fields of biology, biomaterials, mechanical engineering, and 3D bioprinting. The ultimate goal is to be able to create custom tissues and organs by laying down suitable biomaterials layer by layer. This could allow for the production of organ models tailored to individual patient needs, potentially leading to personalized medicine.

The materials used in 3D printing can be categorized as hard or soft. Hard materials include thermoplastic polymers, ceramics, and metals, while soft materials include hydrogels and hydrophilic polymers. Soft materials are capable of absorbing large amounts of water and can promote the formation of new tissues due to their permeability to nutrients.

This review aims to evaluate two hard and four soft substances, considering their characteristics and previous uses, particularly in veterinary medicine, to determine their suitability for 3D-printed protective lenses or for enhancing existing lenses with micro- or nano-chambers for controlled and programmed drug release [1,2].

## 2. Hard Materials

### 2.1. Polycaprolactone

PCL (ε-caprolactone) is a synthetic polyester polymer that has garnered considerable attention due to its great potential in biomedical applications. Among synthetic polymers, PCL stands out as one of the easiest to process and manipulate into various shapes and sizes thanks to its low melting temperature and superior viscoelastic properties. It boasts excellent mechanical properties, such as rubberiness, making it easy to modulate, and degrades slowly over several months to years [3]. PCL also exhibits good biocompatibility and bioactivity and has been approved by the Food and Drug Administration (FDA) as non-toxic, allowing for its use in various human applications, including sutures, micro- and nano-devices for drug delivery, and adhesion barriers [4,5].

**Figure 1:**
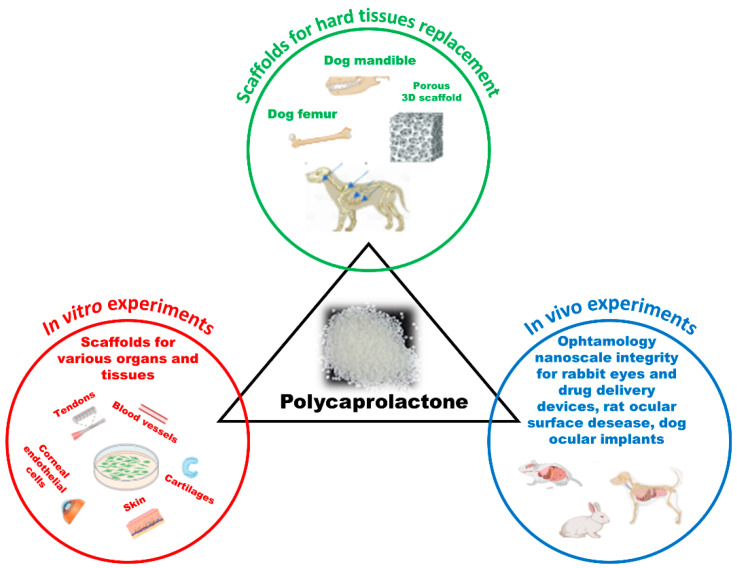
Schematic illustration summarizing common uses and in vivo experiments in veterinary medicine involving PCL (created by BioRender.com).

PCL has found extensive use as a scaffold in tissue engineering for bone, cartilage, tendon and ligament, blood vessels, and skin reconstruction (Figure 1). Its characteristics have made it the ideal material for the fabrication of scaffolds aimed at regenerating hard tissues, such as the femurs of goats [6], repair of partial sternal defects [7], scapula cortical bone removal [8], and mandible defects in dogs [9]. Studies have shown that PCL demonstrates good bone regeneration performance in dog models [10].

PCL has also gained interest in ophthalmology for the development of ocular implants and drug delivery systems (Figure 1). Bernards et al. showed that micro- and nano-engineered PCL can retain its structural conformation and integrity when placed in the eye, marking an important development in the field [11]. Irani et al. demonstrated that PCL is a versatile material. It has been successfully used for drug delivery and in in vitro studies, including those carried out on corneal endothelial cells of bovine [12,13] and humans [14,15,16,17]. These studies have documented the remarkable potential of PCL in the field of tissue engineering. In rat eyes, PCL has shown the ability to be loaded with growth factors and promote the regeneration and growth of ocular epithelial cells. It can also remain attached to the cornea, suggesting its potential use in the treatment of ocular surface disease [18]. In rabbits, PCL drug delivery devices containing hypotensive [19] or antimetabolite [20] agents are biocompatible and efficiently distribute the drug in ocular tissues [11]. Furthermore, in dogs, PCL custom-made prostheses and ocular implants developed using 3D-printing technology have yielded positive results. The artificial eye was aesthetically pleasing, and its use has not led to significant complications.

### 2.2. Pluronic

Pluronics are an important class of biomedical polymers that undergo a reversible gel–sol transition in aqueous solutions at physiological temperature and pH [21,22]. This transition is influenced by the molecular weight and concentration of each polymeric constituent. Pluronics are commonly used in tissue engineering, although they have the drawback of degrading quickly in vivo. To address this, they are often cross-linked with other substances such as α-hydroxy or amino acids to modify their chemical structure.

**Figure 2:**
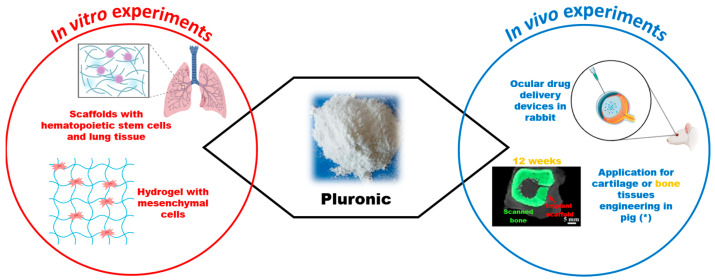
Schematic illustration summarizing common uses and in vivo experiments in veterinary medicine involving pluronic (adapted from https://doi.org/10.3390/ma15051971).

In terms of applications, pluronics are known to inhibit surface-tissue adhesion for many cell types [22]. They have been successfully used in scaffolding applications involving in vitro hematopoietic stem cells and lung tissue [23]. Additionally, various studies showed that pluronics can serve as a potential drug delivery system [24,25,26] and have applications in rabbit ophthalmology [27] (Figure 2).

Among these polymers, pluronic F-127 (poloxamer 407) is a synthetic hydrogel consisting of units of ethylene oxide (PEO) and polypropylene oxide (PPO) that has been approved by the FDA for drug delivery applications in recent years [28]. It has good properties: it is non-toxic, biocompatible, and biodegradable; it has a reversible mechanism of gelation [29] and is thermosensitive. This property enables it to hold encapsulated cells in its structure and to promote initial cell adhesion inside the defect site [30,31]. Moreover, pluronic F-127 can enhance cell attachment, collagen formation, and angiogenesis [32,33].

Additionally, in vitro studies have documented that pluronic F-127 hydrogel is a good substance for tissue engineering [34], as it can be used for the immobilization of dental mesenchymal cells and the healing of cartilage or bone tissues in pigs [35] (Figure 2).

Unfortunately, to our knowledge, pluronic has not been specifically used in the ophthalmic field. However, its biocompatibility, ease of preparation, mechanical stability, antibacterial effect, and ability to incorporate different substances with pharmacological activity and promote their release [36] make it a matrix that should also be investigated for ophthalmological drug delivery use.

## 3. Soft Materials

### 3.1. Silk

Silk is a biopolymer consisting of two distinct proteins, fibroin and sericin. In Bombyx mori cocoons, fibroin makes up about 70 to 80 wt.% and is commonly used in the textile industry and medicine after degumming [37]. The high mechanical strength of fibroin is due to the antiparallel alignment of β-sheets in its protein structure, as well as its hydrophilic and hydrophobic blocks in a semi-crystalline polymer matrix, self-cooling ability, and lack of inflammatory responses in humans [38]. Sericin is also used in biomedical systems for its high moisture, oxidation resistance, and protection against UV radiation [39]. Both silk proteins have been utilized to enhance the physical properties and biocompatibility of various materials in different ways and forms (e.g., in vivo modification, regeneration, or post-treatment).

**Figure 3:**
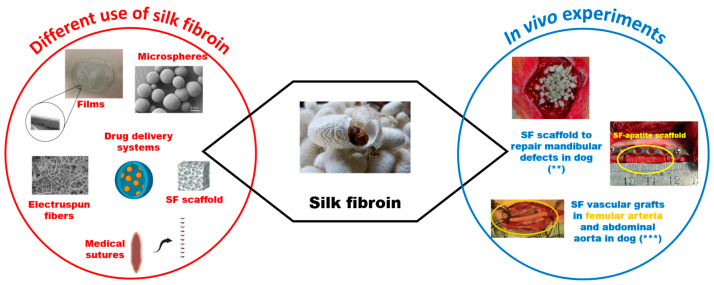
Schematic illustration summarizing common uses and in vivo experiments in veterinary medicine involving silk (adapted from https://doi.org/10.3390/ma15051971).

Silk fibers (SFs) are employed to create various dimensional systems, such as films, nano- or micro-spheres, or electrospun fibers [40]. This is feasible because silk possesses high mechanical strength, controllable degradation, manufacturing flexibility, and good biocompatibility [41,42]. Consequently, it is primarily used for biological applications, such as medical sutures, tissue regeneration [43], drug delivery systems, and for designing biosensors and wearable electronics [44,45,46] (Figure 3).

SF has been studied for the production of various biomaterials for wound healing, such as films, nanofibrous matrices, and 3D porous scaffolds. SF has been used alone or combined with other biomaterials, like polyethylene glycol, keratin, and collagen. It has also been bio-functionalized for wound repair, stabilization of molecules, maintenance of bioactivity, and drug delivery systems [47,48]. SF has excellent properties as a drug carrier, enabling delayed release in therapeutic protocols.

The excellent biomodulating properties of SF make it a great substrate for bone tissue engineering applications. In vivo studies showed SF osteogenic potential in rats [49]. SF scaffolds have been successfully used for repairing bony defects, such as canine mandibular border defects [50]. Composite scaffolds with osteogenic potential and the ability to mimic the natural bone environment were created by combining SF with other biomaterials like hyaluronic acid. SF scaffolds can be produced in different forms as follows: injectable and printable gels, porous sponges, and electrospun 2D and 3D constructs.

SF has also been tested in the vascular field. Since the implantation of artificial SF vascular grafts in the femoral arteries of dogs, the high patency and remodeling ability of these SF grafts have been documented [51], which could be applied in small-diameter (<6 mm) vessels. The implantation of SF vascular grafts in the abdominal aorta of dogs has shown rapid endothelialization and a tendency to form thin luminal layers [52] (Figure 3).

While not extensively tested in the ophthalmic field like pluronics, SF’s versatility and biocompatibility with both hard and soft tissues make it suitable for use in this field for the creation of protective lenses for veterinary use or the functionalization of existing lenses.

### 3.2. Collagen

Collagen is a key component of the extracellular matrix found in various connective tissues, such as bone, cartilage, cornea, veins, arteries, and skin. It helps maintain tissue integrity [53], provides transparency to the cornea and crystalline lens of the eye, and is primarily composed of collagen type I and collagen type IV [54]. Collagen is widely used in corneal bioengineering due to its safety, flexibility, biocompatibility, biodegradability, and low antigenicity. It can form a transparent colloidal solution, and collagen-based nanoparticles are used for topical drug release. However, a drawback of collagen is its lack of mechanical toughness and elasticity, but research has focused on addressing this through collagen cross-linking [55].

The biocompatibility of human collagen type IV has been demonstrated in dogs since the 1980s with intracorneal implants [56], and animal-derived collagen has been utilized for scaffold fabrication and biocompatibility evaluation [53].

Collagen has been used for shields, lenses, hydrogels, and keratoplasty. Collagen shields have been used for ocular surface protection in humans and rabbits in the case of corneal wounds [57]. It has been demonstrated that the collagen shield is a useful drug reservoir because it can prolong the contact time between the cornea and the substance and promote drug delivery to the eye. Many studies showed that collagen shields could be easily used to deliver antibiotics, antivirals, analgesics, and immuno-suppressive drugs to the eye. Collagen shields were effective in delivering tobramycin, fluoroquinolones, cyclosporine, and eplerenone to the eyes of rabbits [58,59,60,61,62]. In a mouse model, collagen discs effectively released and reduced viral replication [63].

Several studies in animal models showed that cross-linking collagen used for corneal lens transplantation can significantly enhance corneal biological and mechanical properties, increasing corneal resistance to tension [64,65]. Recently, the antibiotic release capacity of anionic collagen/polyvinyl alcohol membranes was found to be superior to soft contact lenses and collagen shields. These findings suggest that collagen/polyvinyl alcohol membranes would improve the treatment of corneal lesions in domestic animals, increasing patient welfare [66].

Collagen hydrogels are considered a promising method for corneal wound healing. These hydrogels can support cell growth, facilitate gas exchange, release nutrients and drugs, and remove waste products [67]. In guinea pigs, collagen was safely used for implanting gel into the cornea [68]. In a rabbit experimental model, type I collagen hydrogel with azide and dibenzocyclooctyne successfully promoted corneal re-epithelization [69]. A collagen-based hydrogel loaded with a neuro-regenerative drug effectively replaced a large corneal defect in rabbits, also promoting nerve regeneration [70]. Additionally, cross-linked collagen gel can be used to produce 3D structures ideal for corneal cell growth [55].

**Figure 4:**
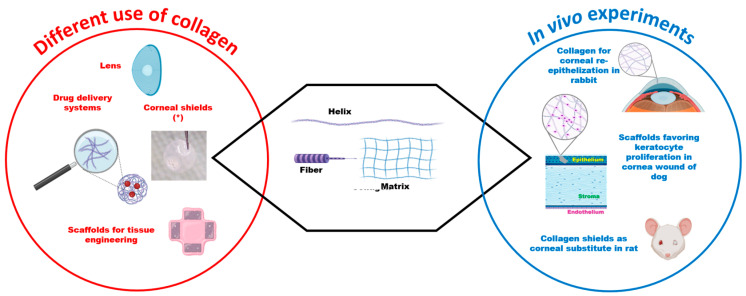
Schematic illustration summarizing common uses and in vivo experiments in veterinary medicine involving collagen (adapted from https://doi.org/10.3390/ma15051971).

Bioengineered corneas should closely resemble natural corneal structures. In a rabbit model, stabilized recombinant human collagen-phosphorylcholine implants promoted corneal cell and nerve repopulation in cases of corneal damage caused by alkali exposure. It has been demonstrated that enzyme-resistant biosynthetic substitutes for allogeneic tissue may be a valid alternative for cases requiring treatment by keratoplasty [71]. An acellular non-cross-linked collagen-based scaffold was transparent, non-immunogenic, and biocompatible for anterior lamellar keratoplasty in a rabbit model [72] (Figure 4).

Atelocollagen, a type of collagen with low antigenicity, has previously been used for treating skin and mucous membrane diseases. In dogs, atelocollagen has been used as a scaffold for keratocyte proliferation, promoting re-epithelization and accelerating corneal wound healing without rejection and inflammation [73].

### 3.3. Alginate

Alginate is a polysaccharide composed of β-D-mannuronic acid (M block) and α-L-glucuronic acid (G block) blocks. Alginate with a high M block is more flexible and elastic but also more immunogenic [74]. Commercially available alginate is obtained by treating the cell walls of brown algae (class Phaeophyceae) with sodium hydroxide. The molecular weight of available alginate varies from 32,000 to 400,000 g/mol. Alginate with high molecular weight shows better physical and biological properties. Alginate has several advantages: it is non-toxic, biodegradable, transparent, low immunogenic, inexpensive, and rapidly gelling [74]. Alginate is an ideal drug carrier due to its mucoadhesiveness and penetration properties [75].

Alginate is an interesting biomaterial useful for regenerative medicine because it promotes cell growth and exhibits significant cross-link ability and biocompatibility. Alginate can be used with other biological components to promote cellular growth and adhesion [74]. Unfortunately, alginate hydrogels dissolve uncontrollably, release alginate strands, and are unable to endure heavy loads due to their poor mechanical strength and high swelling rate [76]. Furthermore, alginate with high molecular weight is slowly metabolized by mammals, but the sodium periodate oxidation of alginate allows it to degrade in a controlled manner [77].

Alginate is usually combined with various biomaterials to improve biomechanical properties for producing tissue-like devices. Alginate constructs combined with gelatin, cellulose, silk, and hyaluronic acid have been successfully used for 3D-printed multilayered structures for long-term culture [74].

Alginate has been used in various cell delivery-based approaches for corneal repair. Oxidized alginate gels have served as useful corneal wound healing bandages. In situ, alginate/chitosan hydrogel has been employed as a limbal stem cell transplanting scaffold for corneal reconstruction following serious corneal alkali burn wounds in rabbits [78]. Another in situ forming composite non-toxic, histocompatible, and rapidly biodegradable hydrogel based on sodium alginate dialdehyde and chitosan was able to reconstruct the engineered corneal endothelium in rabbits [79]. Alginate has been recently used to produce ion-activated bioadhesive hydrogel composed of natural corneal extracellular matrix. Alginate enabled ion-activated hydrogel desirable transparency, biocompatibility, and robust adhesion. This transparent hydrogel, combined with a soft contact lens, rapidly restored normal corneal curvature, allowed for fast corneal re-epithelization, and promoted nerve regeneration [80].

Alginate may be employed as an ocular delivery system, either alone or in combination with other biomaterials, thanks to its mucoadhesiveness, penetration enhancer, and gelification properties, which allow for predictable drug release [74]. Alginate-based multilayers are widely used to control drug release from ophthalmic lenses in humans [81,82]. In rats, thiolated chitosan prepared with sodium alginate nanoparticles delivered large amounts of drugs into the cornea [83]. In rats and mice, alginate-gelatin hydrogel-loaded nanoceria was effective in preventing choroidal neovascularization, neurodegeneration, and protecting the retina from oxidative damage [84].

Numerous studies have explored the use of alginate as a drug delivery system in rabbits. It has been observed that ophthalmic alginate gels and films increased the ocular miotic response compared to pilocarpine drops [85]. Two experimental designs optimized an ophthalmic in situ gelling method to deliver moxifloxacin for treating various ocular infections, ensuring drug release for up to 12 h without local side effects in rabbits [86,87,88]. Furthermore, a multilayered sodium alginate-chitosan hydrogel encapsulated timolol maleate and levofloxacin, serving as a drug delivery system for the treatment of experimentally induced glaucoma in rabbits [89]. Alginate has also been used as a drug delivery system to treat bacterial keratitis. For instance, alginate coated with polycaprolactone/polyethylene glycol fibrous inserts increased the adhesion of the besifloxacin complex [90].

Notably, alginate administered orally could be a useful treatment for certain ophthalmic diseases. For instance, alginate oligosaccharide, administered by gastrogavage for four weeks, prevented experimentally induced cataracts in C57BL/6J mice by reducing oxidative damage [91].

In addition to its medical applications, alginate can also be used for the storage and transport of various cellular types (e.g., human corneal epithelial cells) [92].

**Figure 5:**
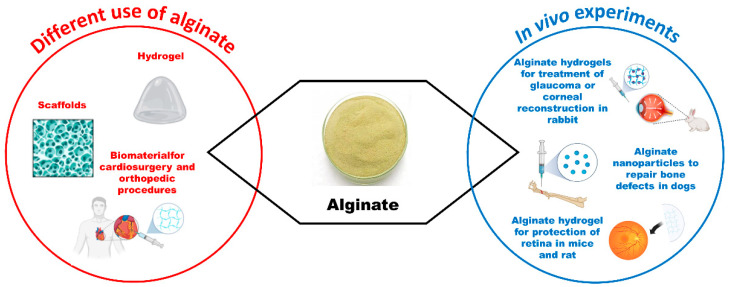
Schematic illustration summarizing common uses and in vivo experiments in veterinary medicine involving alginate (created by BioRender.com).

Alginate has been experimentally utilized in animals as a biomaterial for cardiosurgery, orthopedic procedures, and the treatment of endocrine disorders. Sodium alginate was impregnated into a porous polyester vascular graft, which was successfully implanted in the aorta of mongrel dogs [93]. In dogs, alginate has been employed for mesenchymal stem cells and osteoblast cultures for use in the repair of bone defects [94]. Additionally, alginate combined with poly-L-lactic acid has been used to produce a specific porous scaffold for the repair of osteochondral defects in the canine vertebrae. This system exhibited good osteointegration combined with new bone tissue formation and no inflammatory side effects [95] (Figure 5). Furthermore, chitosan-alginate capsules were found to be safe and biocompatible when used for xenogeneic and allogeneic islet transplantations in a canine model of diabetes [96].

### 3.4. Hyaluronic Acid

**Figure 6:**
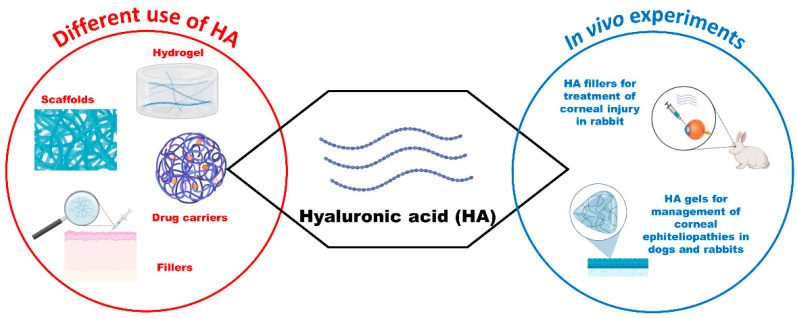
Schematic illustration summarizing common uses and in vivo experiments in veterinary medicine involving hyaluronic acid (created by BioRender.com).

Hyaluronic acid (HA) is a natural non-sulfated polyanionic polysaccharide found in the extracellular matrix of various tissues [97]. It possesses biodegradable, biocompatible, atoxic, viscoelastic, and bioadhesive properties, with a molecular weight ranging from 1000 to 10,000,000 Da. HA plays a crucial role in cell attachment, migration, differentiation, development, and angiogenesis. It can regulate intracellular signaling and cell behaviors through interaction with specific cellular receptors [98]. Clinically, HA can be used for tissue regeneration and cell therapies. It can be used as tissue fillers, drug carriers, or tissue engineering scaffolds in medical specialties, such as wound healing, cartilage tissue repair, and ophthalmology (Figure 6).

Due to its viscoelastic and hydrophilic properties, HA is commonly used as a lubricant in artificial tears for treating dry eyes and accelerating healing after surgery or trauma by binding with corneal epithelial cell CD44 receptors. Furthermore, HA reduces inflammatory mediators and improves the protection of cells from oxidative damage [75,99]. HA hydrogel reduces inflammation and can be used as a regenerative scaffold to accelerate wound and corneal healing [100]. In corneal injury, HA served as a component of a tissue filler material promoting corneal epithelial cell growth without hyperplasia and stromal myofibroblast formation in a rabbit model [101]. Additionally, studies showed that HA aids in the healing and quality of corneal lesions, as well as being a successful physical barrier in the therapy of corneal epitheliopathies in rabbits, dogs, cats, and horses [102,103,104,105]. HA/chitosan/gelatin hydrogel has been shown to promote rapid corneal re-epithelization in a rabbit model of alkali-induced corneal damage [106]. Furthermore, the recovery of normal corneal endothelium has been demonstrated after the transplantation of HA cell-loaded hydrogels to rabbits with corneal endothelium dysfunction [107].

In ophthalmic surgery, HA is employed in cornea tissue engineering due to its biological stability, biodegradability, and permeability of nutrients. However, its low stability may pose drawbacks in cornea tissue engineering [75]. HA can establish and maintain comfortable conditions to promote healing of the postsurgical area, minimize the risk of adhesions, decrease oxidative damage, and normalize intraocular pressure [108]. Studies have also shown that HA-based microcarriers enhance corneal stromal regeneration in a rabbit model of corneal alkali burn injury, achieving corneal healing after intracorneal injection of keratocytes/functionalized HA-based oxidized microcarriers [109].

Moreover, HA is utilized as a stem cell culturing system, and it enhances stem cell proliferation. Nanofibers of HA scaffolds are used to support or grow mesenchymal stem cells directly on them. The introduction of different cross-linking networks has also allowed HA gels to be more conducive to stem cell differentiation [108].

Numerous studies have focused on the use of hyaluronic acid (HA) scaffolds for corneal healing, particularly as cell delivery vehicles. Porcine stem cells loaded into the HA hydrogel vehicle showed promising differentiation, adhesion, and proliferation abilities. Additionally, HA hydrogel loaded with dopamine demonstrated improved adhesiveness and increased cell viability [110]. In in vivo studies on rabbits, the cornea implant surface was enhanced with different molecular weights of HA, leading to a significant increase in the number of keratocytes [75].

It has been demonstrated that biocompatible HA hydrogels with large microporosities can be effectively used as scaffold systems for the treatment of various endothelial corneal dysfunctions because they allow for nutrient permeation [109]. An in vivo study reported that implanting endothelium cells/HA devices in the anterior chamber was clinically suitable for treating corneal wounds but might cause some inflammatory side effects [111]. Highly oxidized cell/HA systems successfully restored the physiological collagenous structure after 4 weeks in a rabbit model. It is well known that oxidation promotes cell proliferation and adhesion, facilitating a more rapid restoration of physiological tissue conditions. Furthermore, HA microgels may be useful systems for bioactive delivery, injectable fillers, and 3D bioprinting [109].

Drug delivery through soft contact lenses (SCLs) is a feasible method. HA is safely used in the structure of silicone SCL without affecting the optical properties. HA promotes physiologic blinking, increases drug residence time on ocular tissue by reducing tear outflow, and prevents protein adhesion to the SCL surface. An in vivo study in a rabbit model with dry eye syndrome demonstrated that SCL released HA into the rabbit eyes for 2 weeks, promoting fast healing [112]. Poly (2-hydroxyethyl methacrylate)/β-cyclodextrin-HA hydrogel has proven to be useful as an SCL material for conjunctivitis treatment in rabbits. These SCLs showed good oxygen permeability and flexibility, reduced the adhesion of Staphylococcus aureus, and enhanced drug delivery [113].

SCL constructed with HA and loaded with ciprofloxacin and dexamethasone released an adequate amount of antibiotic [114]. The soaking technique and direct entrapment were tested to load HA in SCLs. In an in vivo study in rabbits, direct entrapment was superior to the soaking method in terms of HA quantitative release and residence times [115].

HA can be cross-linked or conjugated with various biomaterials for controlled-release formulations, and it can effectively encapsulate many drugs, even at the nanoscale [116]. Some ionic complexes between HA and various drugs have been shown to prolong ocular residence time. The advantageous rheological and mucoadhesive properties of HA loaded with 0.5% timolol prolonged the drug’s residence time, preventing its removal due to blinking in normotensive rabbits [97]. Moreover, HA has been used in producing long-lasting ciprofloxacin and vancomycin release systems for postoperative therapy in ophthalmic surgery [117]. It was also combined with β-cyclodextrin to develop a delivery system loaded with corneal epithelial cells and dexamethasone [118]. Its carrier capacity has been demonstrated when conjugated with gold; in fact, HA increased the mobility of the gold nanoparticles and favored their binding to HA receptors in various cells of the porcine eye [119]. HA has been used to fabricate pliable eye bandages containing biodegradable microneedles for targeted ophthalmic medication administration in rats [120].

## 4. Conclusions and Future Perspectives

The interest in biomaterials among researchers is continuously increasing. Many studies focus on therapeutic solutions for human beings, but research in veterinary medicine also aims to improve animal welfare.

This review summarizes the previous applications of various biomaterials in experimental, pre-clinical, and clinical studies, particularly in veterinary medicine and ophthalmology.

Some biomaterials, such as collagen and hyaluronic acid, are basic structural components of most tissues and play an essential role in maintaining the biological and structural integrity of the tissue architecture. Most biomaterials are easy to handle and could be used in tissue engineering. To encourage the clinical application of these systems, it is necessary to optimize production to provide an adequate imitation of biological functions. Therefore, natural and synthetic biomaterials should ensure a favorable environment for the cells.

The biomaterials examined in this review may be used in various medical areas, including ophthalmology. They can be employed in the form of gels, scaffolds, and 3D constructs and can be safely used as a growth substrate for many cells and as stroma substitutes [121,122,123,124]. The greatest interest is directed towards devices that can be used as drug or cell delivery systems.

Topical administration in the eye is usually based on ophthalmic drops, which require frequent instillation and cause discomfort for the patient. Important goals of future research could be to design biocompatible and well-tolerated SCLs specifically for drug delivery and to identify the most effective biomaterial for this purpose. SCLs are a more natural technique to administer ophthalmic drugs than eye drops, as they are near the cornea [125]. SCLs consist of hydrogel able to absorb a fixed volume of an aqueous vehicle, including drugs and nanoparticles inside a polymerizable monomer solution able to manage the related release and reduce side effects due to systemic absorption.

Therapeutic SCLs for drug delivery may overcome the main drawbacks of traditional eye drops, such as low drug bioavailability, low duration of action of the drug, low patient welfare, frequent drug administration, and systemic toxicity. Moreover, the drug released by the SCLs remains in the tear film for at least 30 min, allowing the drug to achieve therapeutic concentration in most of the cornea, demonstrating that the bioavailability increases to about 50% with SCLs [126].

## Data Availability

No new data were created or analyzed in this study. Data sharing is not applicable to this article.
